# Effects of urban street geometry and traditional kabalti passages on building surface temperature in a hot-dry climate

**DOI:** 10.1038/s41598-025-34532-z

**Published:** 2026-01-07

**Authors:** Şefika Ergin, Kübra Suna Gider, İbrahim Halil Şeker, Hasan Yildizhan, Arman Ameen

**Affiliations:** 1 Department of Architecture, Faculty of Architecture, Dicle University, Diyarbakır, Turkey; 2 Department of Architecture, Faculty of Architecture, Bingöl University, Bingöl, Turkey; 3 Energy Systems Engineering, Engineering Faculty, Adana Alparslan Türkeş Science and Technology University, 46278 Adana, Turkey; 4 Department of Building Engineering, Energy Systems and Sustainability Science, University of Gävle, 801 76 Gävle, Sweden

**Keywords:** Kabaltı, SVF (sky view factor), Thermal comfort, Surface temperature, Urban areas, Climate sciences, Ecology, Ecology, Engineering, Environmental sciences, Environmental social sciences

## Abstract

In hot climate regions, the direct impact of solar radiation on building surfaces, including heat absorption and storage, negatively impacts outdoor comfort and the living conditions of urban residents. This study investigates the impact of urban street geometry on building surface temperatures in a hot and dry climate, focusing on the traditional Suriçi district of Diyarbakır. Measurements were conducted at 25 locations throughout the year along streets with varying sky view factor (SVF) values and within vaulted covered passages (kabaltıs). In the study, a Testo 410-2 anemometer was used to measure air temperature and a thermal camera was used to measure surface temperature. The results show smaller daily surface temperature amplitudes in regions with lower SVF values and in kabaltıs with an SVF value of 0. Measured surface temperatures reached as high as 58.8 °C at high SVF locations, while they remained around 30 °C in shaded kabaltıs. The findings indicate that street geometry parameters such as building height, spacing, and orientation significantly influence microclimate conditions. Differences of up to 15–20 °C were observed between shaded kabaltıs surface temperatures and other surface temperatures at measurement points where the SVF value was close to 1. Reducing SVF through design strategies such as the use of kabaltıs and planting trees can improve outdoor thermal comfort in hot climates.

## Introduction

Urban phenomenon, such as the influence of street geometry on surface temperature, directly impact human life due to the interaction between people and the environment^1,2^. People can experience different thermal conditions when using different urban environments (e.g., streets, plazas, urban parks) under varying microclimatic conditions throughout the day^3^. The frequency and intensity of such outdoor activities influence the comfort levels of urban residents^4^. For example, thermal discomfort experienced by people exposed to the sun on a hot summer day depends on a specific combination of air temperature, the surface temperature of surrounding areas, wind speed, and humidity level^5^. Similarly, the intensity of solar radiation may discourage people from using existing urban spaces^6^. Conversely, in a cold region, a particular combination of wind speed and air temperature, or the provision of sunny areas sheltered from prevailing winds, can encourage outdoor activity. Urban residents are continuously exposed to varying thermal conditions that change with the seasons throughout the year. Assessing people’s perception of thermal comfort in urban areas is therefore important for both urban development and the quality of life of city dwellers^7^.

Climatic conditions in urban environments significantly affect people’s lifestyles and health^8,9^. Identifying the factors that influence thermal conditions in cities and optimizing them at the architectural design scale is crucial for increasing urban resilience against thermal disturbances^10–12^. The increasing migration to urban areas has led to a rapid rise in city populations. According to the United Nations (2019), approximately 70% of the world’s population is expected to live in cities by 2050. Rapid urbanization alters the urban energy balance, causing more heat to be absorbed, produced, and stored, which in turn exposes cities to higher temperatures than surrounding rural areas^13–16^. The urban heat island (UHI) effect is characterized by elevated temperatures resulting from impermeable surfaces and complex urban structures^17,18^. The magnitude of the UHI effect is influenced by urban layout, morphological and structural characteristics, material properties, climatic conditions, anthropogenic heat emissions, and the choice of reference rural measurement station^15,19^. In recent years, many cities have faced the growing threat of extreme heat due to the UHI effect. A global study examining 13,115 cities between 1983 and 2016 revealed that urban exposure to extreme heat increased by nearly 200%, affecting 1.7 billion people^20^. Factors shaping the urban thermal environment also increase building energy demand by raising ambient temperatures^21^. Kolokotroni et al.^22^ reported that a 1 °C temperature increase in urban environments can raise summer cooling energy demand by 5–10%. Rising temperatures not only increase energy consumption but also negatively affect the economy, environmental quality, and the creation of comfortable urban spaces^16^. The urban thermal environment is shaped by both surface albedo-the reflective capacity of materials-and urban geometry, which amplifies radiation absorption through multiple reflections between building surfaces^23,24^. The effect of urban geometry on radiation balance is an important factor contributing to surface temperature variations across urban elements^25,26^. Roofs, walls, and pavements in urban areas are in constant heat exchange with outdoor and indoor air. The net radiation flux absorbed by a building’s wall influences the indoor thermal environment, while the radiation emitted from exterior surfaces contributes to the outdoor microclimate. For this reason, building exterior wall temperature is an important parameter in heat exchange processes and plays a critical role in ensuring thermal comfort^17^.

While street geometry and SVF have been extensively studied in the literature, the thermal behavior of the kabaltıs have not been analyzed through field measurements. Studies in the literature rely on numerical modeling, user feedback, and observational inference rather than surface temperature measurements. This study aims to address this gap by using quantitative data obtained from a long-term measurement period conducted in the historic Suriçi district.

This study investigates the relationship between street geometry and surface temperatures within the traditional urban fabric of the Sur district in Diyarbakır, which has a hot-dry climate. The urban fabric of the district is characterized by variations in building distances, building heights, height differences, and street orientations. The Sky View Factor (SVF) index, which depends on street geometry, was calculated. In addition, vaulted covered passages known as *kabaltı*, a traditional architectural element, were examined. Surface temperatures of building facades were measured at specified points during morning and evening hours, and their periodic and daily variations were analyzed. The study further explores which environmental characteristics most significantly influenced these temperature fluctuations.

## Methodology

### Description of the study area

**Fig. 1:**
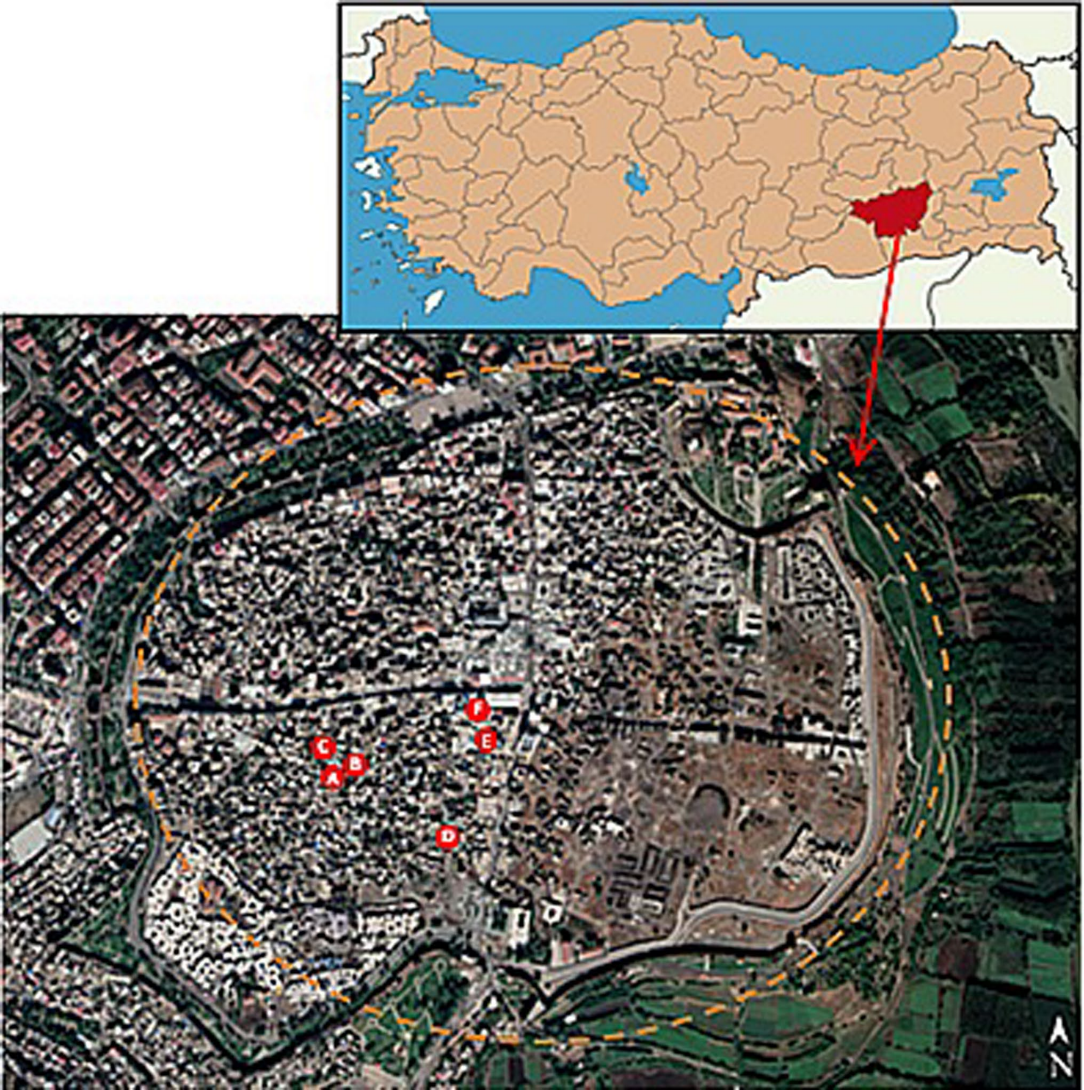
Location of Diyarbakir province on the map of Türkiye and measurement points in the Suriçi region (Measurement points were processed using the Power Point 2016 program on the image taken from Google Maps^28,29^).

The earliest settlement in the city of Diyarbakır began within the inner castle and, for a long time, remained confined to the area inside the city walls due to environmental factors. This led to the development of a dense settlement texture in the Suriçi region (Fig. 1). Over time, as the area within the walls could no longer accommodate the growing population, urbanization expanded outward, primarily toward the northwest^27^.

**Fig. 2:**
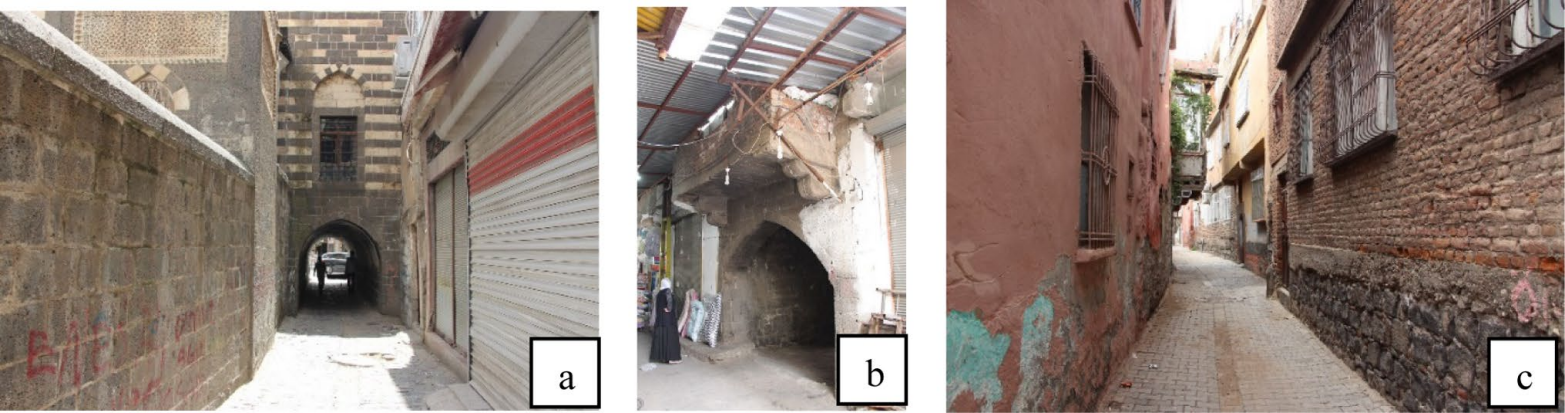
Samples of streets and kabaltıs in Suriçi Region.

In the Suriçi district, alongside traditional low-rise courtyard houses built of basalt stone, more recent high-rise reinforced concrete buildings are also present. The traditional Diyarbakır Suriçi houses, shaped by knowledge accumulated over centuries, were designed with both climatic and socio-cultural considerations in mind. The adjoining courtyard houses collectively formed a distinctive urban fabric (Fig. 2).

The hot-arid climate characteristics of Diyarbakır province make the daily living conditions of the local people difficult. The tall exterior walls, a result of the houses’ introverted spatial organization, create shaded and cool areas along the streets during the summer. As most interior spaces are oriented toward the courtyard, the number of street-facing rooms is limited. In some houses, however, the expansion of street-facing rooms has led to the formation of passages beneath the structures that extend toward the street. These passages, known as *kabaltı*, provide additional shading and produce a cooling effect during the hot summer months (Fig. 2b)^30^.

#### Climate conditions

The city of Diyarbakır, where the study was conducted, is established on 38° latitudes and 40° longitudes. The city, which was established on a basalt plain, is 675 m above sea level. The climate is classified as subtropical highland. According to the Köppen Geiger climate classification, it is seen as Csa. Winters in the region are harsh, while summers are hot and dry. According to meteorological data between 1929 and 2024 in Diyarbakır province; the average highest temperature was 38.4 °C in July, and the average lowest temperature was − 2.2 °C in January. The average sunshine duration was measured in July with 12.4 h, and the highest monthly total rainfall was measured in December with 71.2 mm (Table 1)^31^.

**Table 1:** Climatic metrics of Diyarbakir province for the years 1929–2024^31^.

Metrics	Jan	Feb	Mar	Apr	May	June	July	Aug	Sep	Oct	Nov	Dec	Avg
Average temperature, °C	1,8	3,7	8,3	13,8	19,3	26,1	31	30,5	25,1	17,6	9,8	4,1	15,9
Average highest temperature, °C	6,8	9,2	14,5	20,5	26,6	33,6	38,4	38,3	33,4	25,4	16,4	9,2	22,7
Average lowest temperature, °C	− 2,2	− 1	2,5	7	11,3	16,6	21,7	21,1	16	10,1	4,2	− 0,1	8,9
Average sunshine duration, hour	3,9	4,9	5,6	7,2	9,6	12,1	12,4	11,6	10	7,5	5,5	3,9	7,9
Average rainy day number	12,25	11,32	11,8	11,2	8,73	2,63	0,46	0,32	1,07	5,74	8,19	11,49	85,2
Average monthly total rainfall, mm	69,7	67,2	67,2	68,3	44,4	8,6	1,3	1	5,3	32,5	55,9	71,2	492,6
Highest temperature, °C	16.9	21.8	28.3	35.3	39.8	42	46.2	45.9	42.2	35.7	28.4	22.5	46.2
Lowest temperature, °C	− 24.2	− 21	− 14	− 6.1	0.8	1.8	9.9	11.4	4	− 1.8	− 12.9	− 23.4	− 24.2

#### Field Investigation

There are 9 kabaltıs in the Diyarbakır Suriçi settlement. The kabaltıs in the region change in east–west and north–south orientations. Information about these kabaltıs is shown in Table 2. Basalt stone was generally used as a building material, some of which are bricks and briquettes. There are also examples of walls with plaster applied on basalt stone. Their upper covers are made of wooden beamed flooring, vaults or reinforced concrete. The space above two of them is a part of the mosque structure, and the space above the others is residential spaces.

**Table 2:**
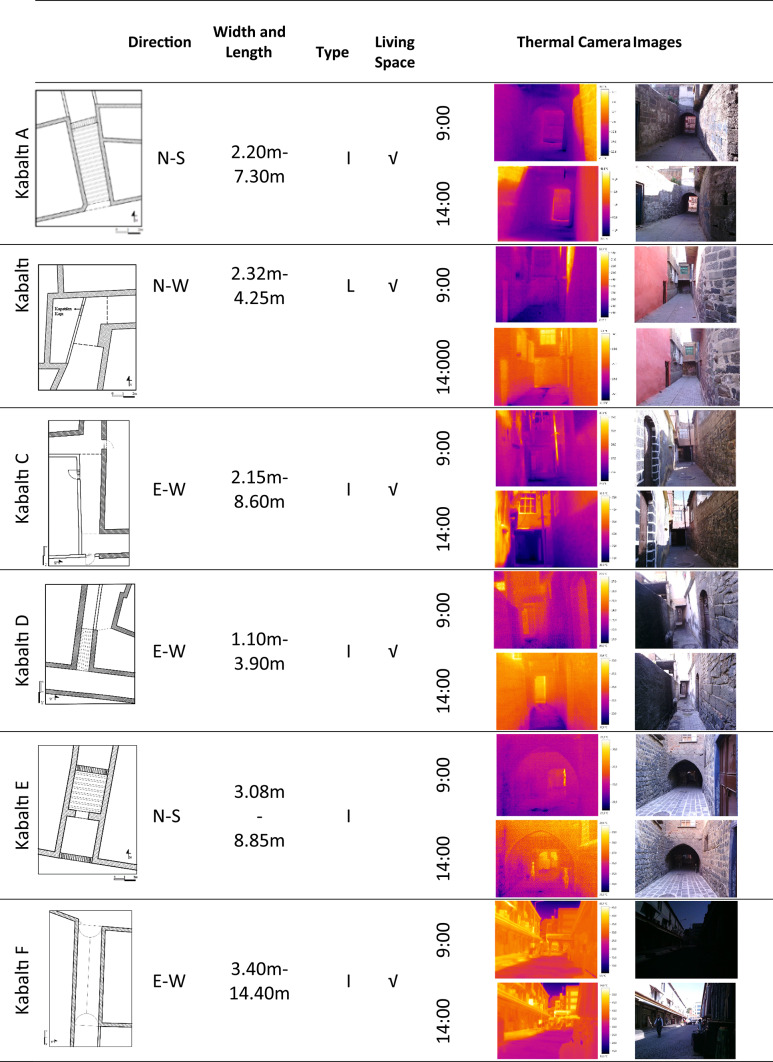
The Kabaltıs in the Diyarbakır Suriçi Region^32^.

There are a total of 9 kabaltıs in the area. Two of these were on the verge of collapse, posing a risk, and one was not studied because it was too far from the work area and difficult to access the measurement clocks. Kabaltıs included in the study are shown on the Suriçi map as “A-B-C-D-E-F” (Fig. 1). A total of 25 points were selected from the 6 kabaltıs in the region and the streets to which the kabaltıs are connected, and measurements were taken from these points. The A-B-C-D-E-E1-F codes indicate the points under the kabaltıs, and the other codes indicate the points taken from outside the kabaltıs. In the selection of the kabaltıs, the fact that they are close to each other in order not to deviate from the on-site measurement time was the determining factor. The kabaltıs A-B-C are in the Ziya Gökalp neighborhood, the kabaltı D is in the Abdaldede neighborhood, and the kabaltıs E-F-G are in the Süleyman Nazif neighborhood.

The heights and distances of buildings surrounding the measurement points, except for kabaltıs, vary (Table 3). Measurement points on streets located between buildings of unequal height are subject to varying levels of shadow.

**Table 3:** Heights and distances of buildings around the measurement points.

Points	Height (m)				Distance (m)
	North	South	East	West	
A1	5.91	3.55	–	–	2.32
A2	–	–	3.02	4.20	2.62
B1	5.60	10.70	–	–	2.50
B2	3.60	5.52	–	–	2.41
B3	–	–	9.90	5.20	2.85
B4	–	–	6.60	6.00	4.63
B5	–	–	5.00	6.00	15.00
C1	–	–	3.85	5.15	2.24
C2	5.90	5.35	–	–	2.27
C3	–	–	22.50	5.95	3.10
D1	1.95	6.81	–	–	1.18
D2	–	–	3.10	3.40	2.45
D3	16.00	2.54	–	–	4.51
E2	–	–	6.00	3.43	3.21
E3	9.35	10.00	–	–	3.95
F1	–	–	7.24	10.24	3.82
F2	0.72	9.00	–	–	2.82
F3	9.00	6.00	–	–	9.00

### Field measurements

**Fig. 3:**
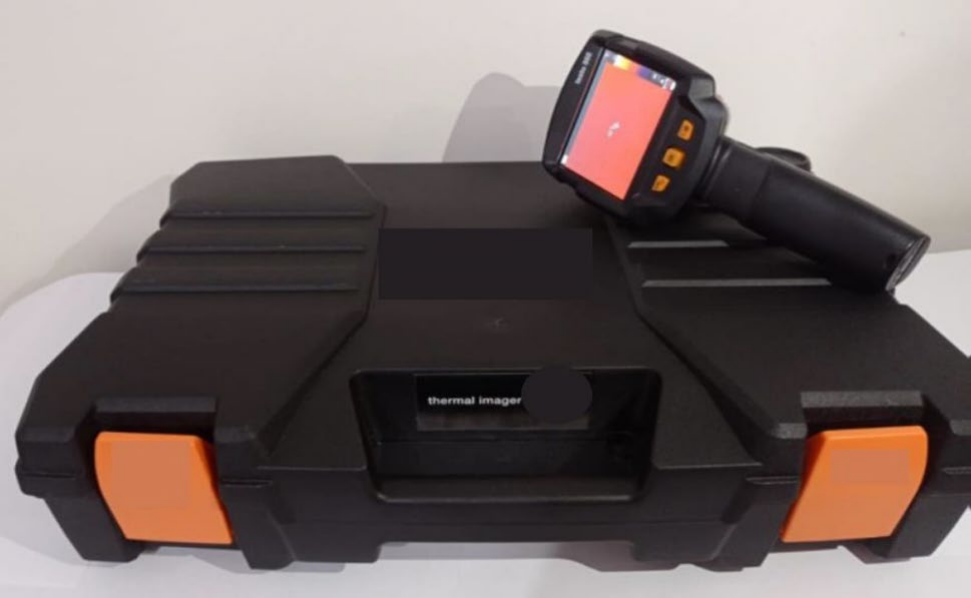
Thermal camera used in the measurement.

In this article, the surface temperatures of the structures on the streets with different geometric features and the kabaltıs used as street elements were examined. The study was carried out in two stages. In the first stage, 6 kabaltı points and 19 points to be measured from the streets connected to the kabaltıs were determined. Measurements were made from one point of the inner wall surface of the kabaltıs. The measured surfaces were constructed from basalt and fire brick. Basalt stone has an emissivity coefficient of 0.72 and fire brick has an emissivity coefficient of 0.75–0.80^33^. Only the surface of two points from the starting and ending points were measured due to the long length of the kabaltı E. Measurements were taken from the wall surfaces in opposite directions of the 25 points determined in total. The SVF values of the points outside of kabaltıs were measured and calculated. The sky photographs used to calculate the SVF values were taken on a clear day with a fisheye lens attached to a Canon EOS 650D brand camera. The photographs were taken by directing the head of the camera to the north and raising the camera 1.5 m from the ground with the help of a tripod. The RayMan pro program was used to calculate the SVF values. In the second stage, the surface temperatures of the buildings were measured with a Testo 868 thermal camera at approximately 10-day intervals between 10/06/2020 and 28/05/2021 from these determined points (Fig. 3). The measurement hours were determined as 09:00–10:00 and 14:00–15:00. There is a time difference between taking measurements from one point and reaching another point to take measurements, and this changes the solar incidence angle. Therefore, each measurement point was measured at the same time on each measurement day with a difference of ± 5 min, taking care to ensure that the measurement times were close. When calibrating the Testo 868 thermal imager, emissivity coefficients were entered manually. These values were based on values from Cole-Parmer. For basalt, they were set at 0.72, while for firebrick, they were set at 0.80. These values were used for each material to ensure accuracy and repeatability of the results. The locations, cross-sections and fisheye photographs of the measurement points are shown in Table 4. In the measurements, the surface temperatures obtained from a north–south oriented street were measured from the east and west walls. The surface temperatures obtained from an east–west oriented street were measured from the north and south wall surfaces.

**Table 4:**
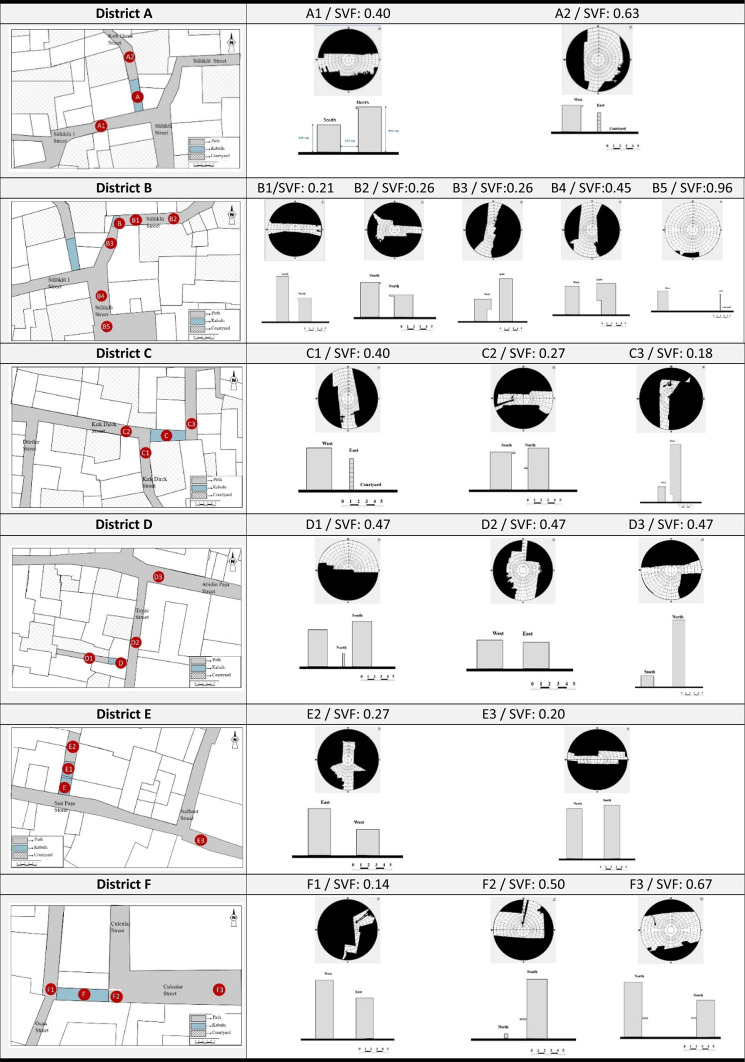
Characteristics of the measured points^32^.

**Fig. 4:**
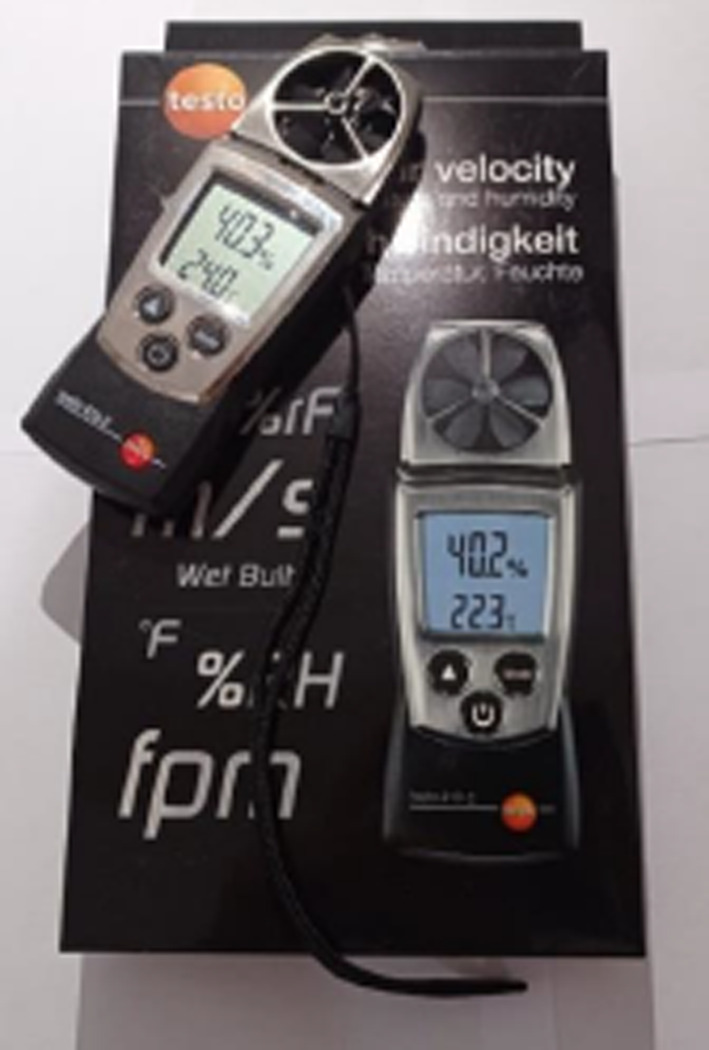
Testo 410-2 anemometer used to measure air temperature.

A Testo 410-2 anemometer was used to measure air temperature in the study (Fig. 4). Air temperature was recorded simultaneously with surface temperature and SVF measurements at each measurement point. The anemometer was positioned 1.10 m above the ground. To provide a reference for the overall thermal status of the surface temperatures at the measurement points, the average surface temperature was calculated. The average surface temperatures were calculated by taking the arithmetic mean of the surface temperature values measured in the morning and midday of the same day for each measurement point. The average surface temperature value presented in the graphs was designed not as an independent analytical variable, but rather as a contextual indicator to support the comparative examination of the thermal status of surfaces affected by different street geometries and kabaltıs.

In this study, to express the effect of SVF values on the daily variation of surface temperature, the diurnal surface temperature amplitude was defined as the absolute difference between the surface temperature values measured in the morning and afternoon at the same front point. The diurnal surface temperature amplitude allowed for the interpretation of the stability of surface temperatures according to the changing SVF values.

## Results and evaluation

**Fig. 5:**
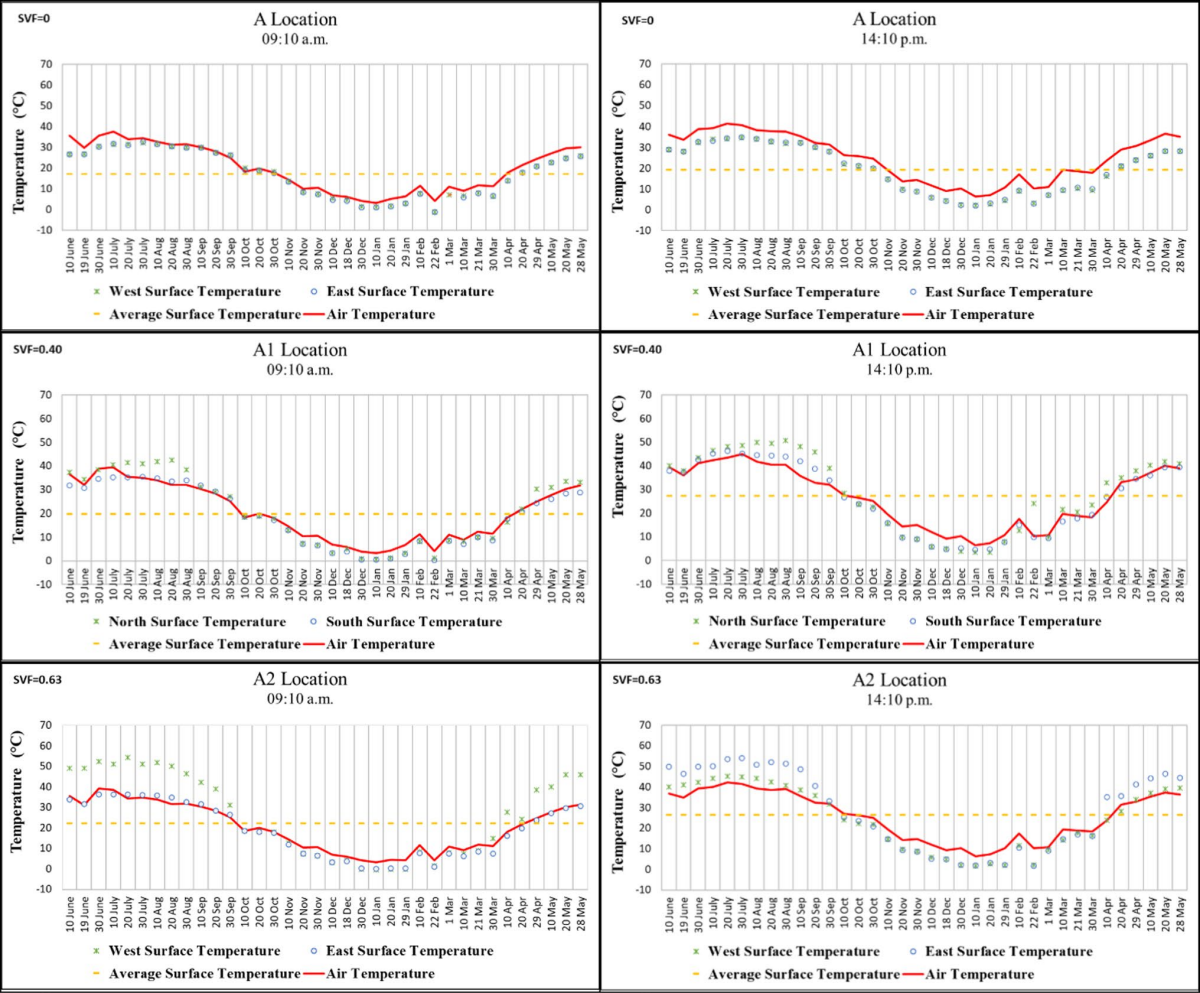
Surface temperature measurement results of location A.

Points A and A2 in Region A are located on a north–south oriented street, while Point A1 is situated on an east–west oriented street. According to the morning measurements taken throughout the year, the highest surface temperature was recorded on 20 July 2021 at 54.3 °C on the west wall of Point A2. The lowest surface temperatures were measured on 22 February 2021, with − 1.2 °C on the west wall of Point A (located under a *kabaltı*) and − 1.3 °C on the east wall. In the afternoon measurements, the highest temperature was recorded on 30 July 2020 at 54 °C, while the lowest was measured on 22 February 2021 at 1.7 °C on the east wall of Point A2 (Fig. 5).

When all data from Point A were examined, the difference in surface temperature between morning and afternoon on the same walls was approximately 1–3 °C. In addition, the east and west wall surface temperatures of Point A were found to be very close to each other. This can be attributed to the SVF value of Point A being “0,” meaning that the building surfaces were not exposed to direct solar radiation. By contrast, the surface temperatures on the same walls at Points A1 and A2 varied by approximately 2–15 °C between morning and afternoon. Due to the higher SVF values at these points, the impact of solar radiation during the afternoon was greater, resulting in larger temperature differences (Fig. 5).

At Point A2, during the summer months and transitional seasons, the west wall was generally warmer than the east wall in the morning, whereas in the afternoon the east wall became warmer than the west wall. In winter, however, the temperature values of the two walls were observed to be similar. These periodic variations at Point A2, which has a high SVF, are influenced by the angle of incidence and the intensity of solar radiation (Fig. 5).

**Fig. 6:**
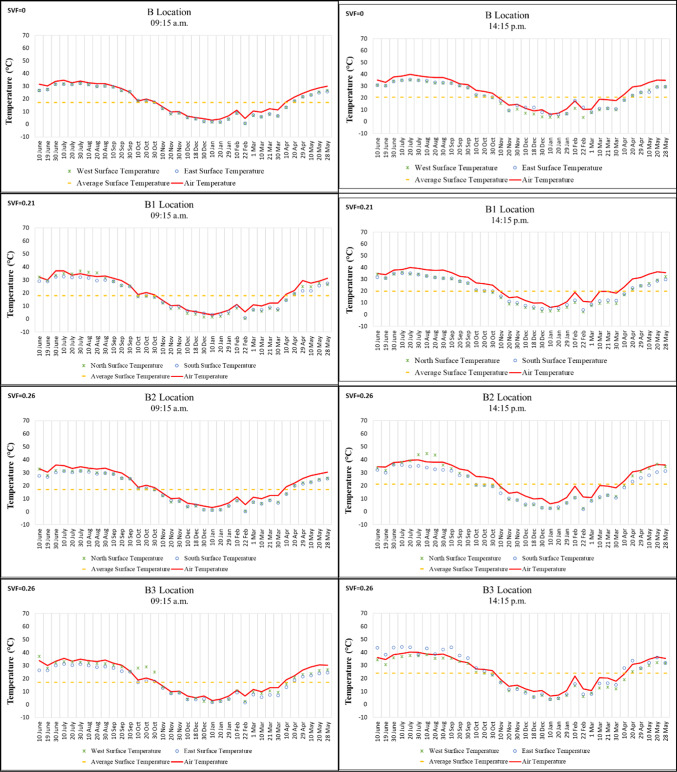

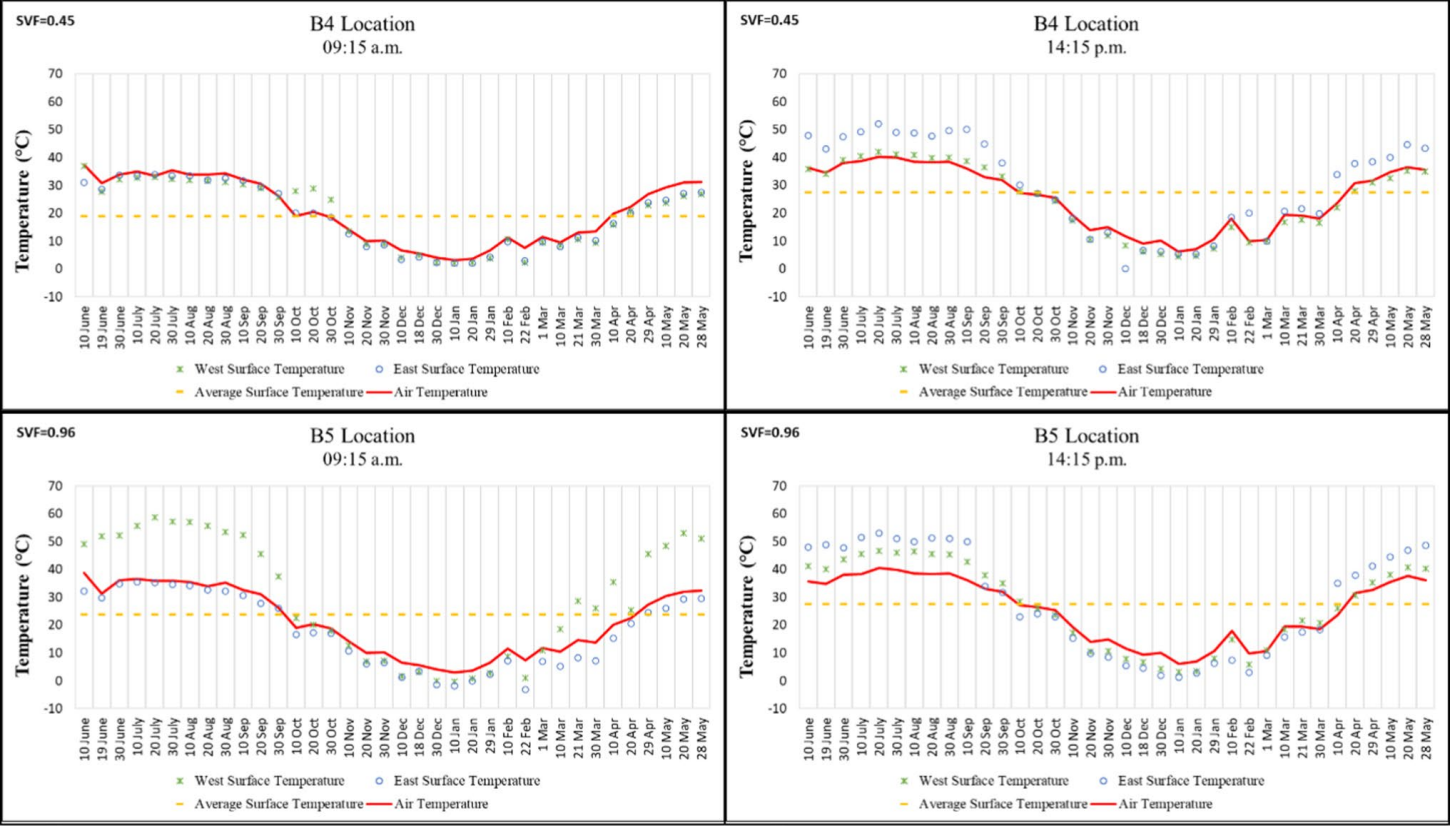
Surface temperature measurement results of location B.

While Points B, B3, B4, and B5 in Region B are located on a north–south oriented street, Points B1 and B2 are situated on an east–west oriented street. In the morning, the lowest surface temperature was recorded at − 3.1 °C on 22 February 2021 on the east wall, while the highest was 58.8 °C on 20 July 2020 on the west wall of Point B5, which has the highest SVF value. In the afternoon, the lowest temperature was measured at 1.2 °C on 10 January 2021 on the east wall, and the highest was 53 °C on 20 July 2020 on the east wall of Point B5 (Fig.6).

Surface temperature differences between morning and afternoon on the mostly shaded building surfaces at Points B1, B2, and B3, each with low SVF values, ranged from approximately 2–5 °C. By contrast, the high SVF value of Point B5 caused its building surface temperatures to be approximately 10–20 °C higher than those of the other points during the hottest period of the year. These findings support previous studies emphasizing the importance of reducing SVF. Feng et al.^17^, for example, confirmed that afforestation and other shading elements reduce the amount of solar radiation reaching building surfaces during the daytime and provide a cooling effect (Fig. 6).

It was also observed that, in winter, the surface temperatures at Point B5, which has a high SVF value, fell below 0 °C, a condition not detected at the other points. This can be explained by the fact that airflow at Point B5 was not obstructed, making it an important contributing factor (Fig. 6).

**Fig. 7:**
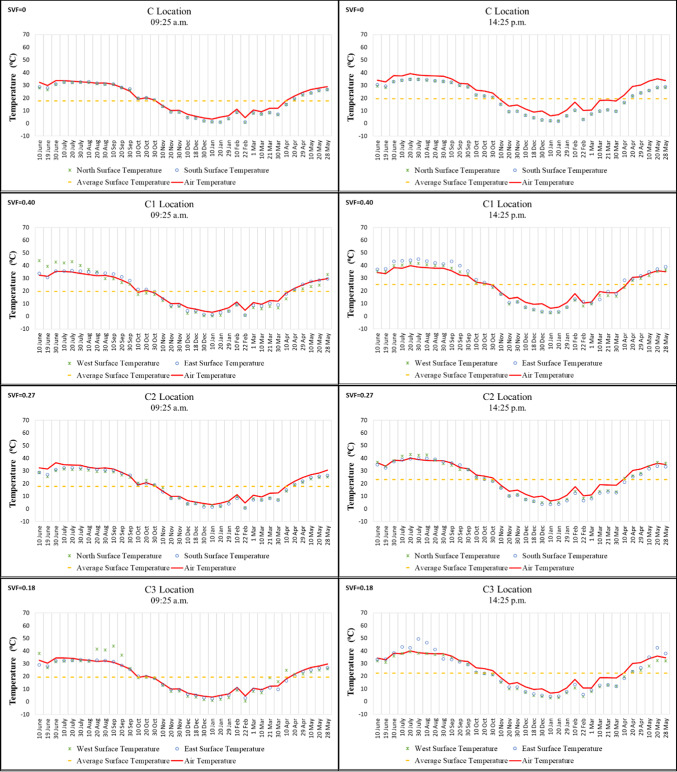
Surface temperature measurement results of location C.

In Region C, Points C and C2 are located on an east–west oriented street, while Points C1 and C3 are on a north–south oriented street. According to the morning measurements, the highest surface temperature was recorded at 44 °C on 10 June 2021 on the west walls of Points C1 and C3, while the lowest was 0.3 °C on 10 January 2022 on the west wall of Point C1. In the afternoon, the highest surface temperature was 49.4 °C on 30 July 2021 on the east wall of Point C3, and the lowest was 1.7 °C on 20 January 2022 on the south wall of Point C. The high temperatures measured at Point C3, despite its low SVF value, can be explained by the differential absorption of reflected solar radiation caused by large height differences between the surrounding buildings. This finding is consistent with Yang and Li^25^, who showed that greater building height differences influence sunshine duration and surface heating. The surface temperatures of the north and south walls of Point C, which has an SVF value of “0” and is not directly exposed to solar radiation, were very similar at all measurement times. The diurnal temperature variation at Point C was also generally smaller compared to other points. By contrast, at Points C1, C2, and C3, the differences between building surfaces exposed to solar radiation reached up to approximately 15 °C. This variation is related to both high SVF values and building height differences. When surface temperatures were compared, the north and south walls of Points C and C2 showed similar values in the morning. However, by noon, the surface temperature of Point C was approximately 4–5 °C lower than that of Point C2. This difference occurred because Point C was covered and therefore not directly exposed to solar radiation. Finally, the surface temperatures of the west walls of Points C1 and C3, which were exposed to solar radiation in the morning, showed little change in the afternoon (Fig. 7).

**Fig. 8:**
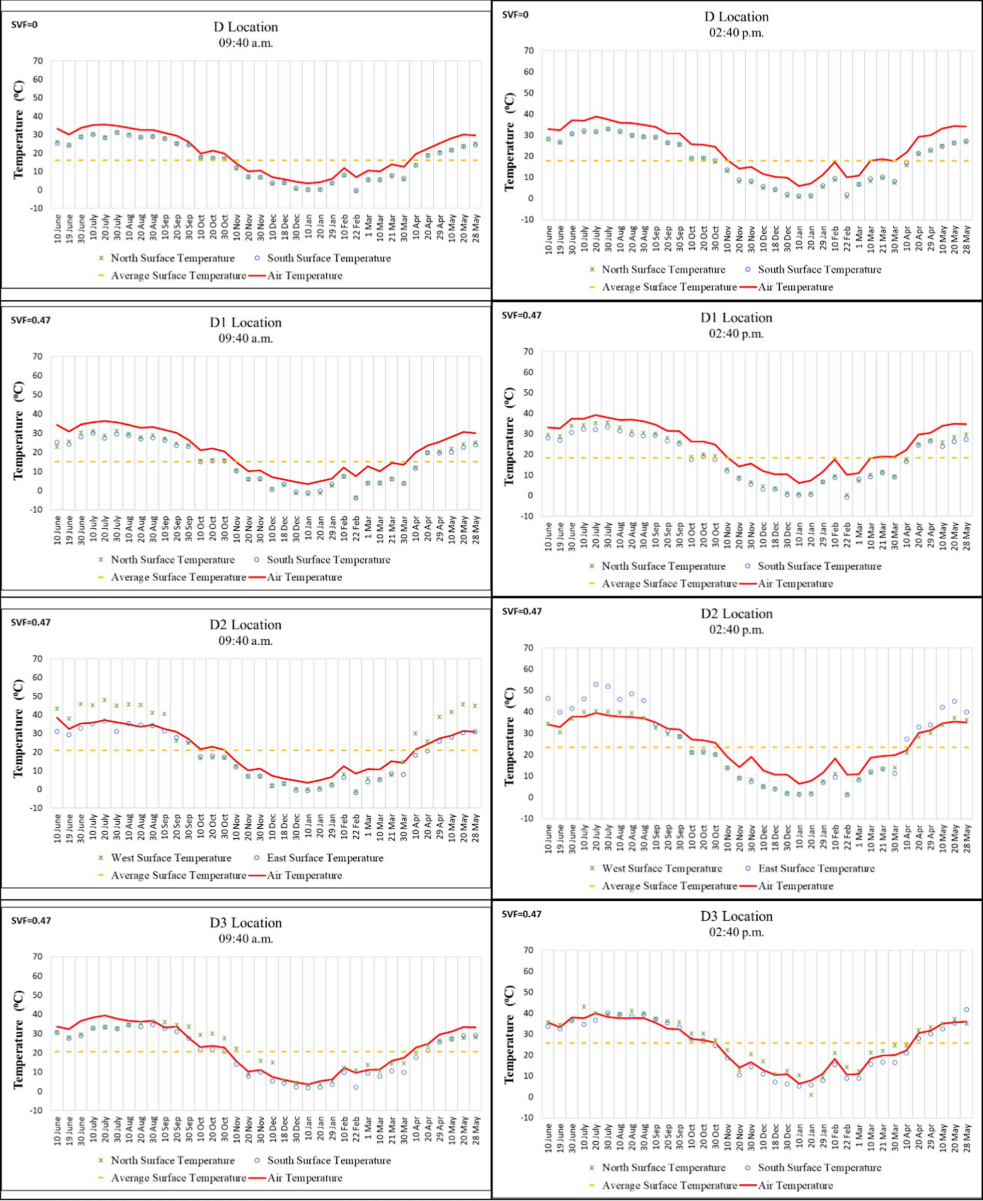
Surface temperature measurement results of location D.

Points D, D1, and D3 in Region D are located on an east–west oriented street, while Point D2 is situated on a north–south oriented street. According to the morning and afternoon measurements, the highest surface temperatures were observed at Point D2 on 20 July 2020. These values were recorded as 48.1 °C on the west wall in the morning and 53 °C on the east wall in the afternoon. The lowest surface temperatures were measured on the south wall of Point D1 on 22 February 2021, with − 3.9 °C in the morning and − 0.6 °C in the afternoon (Fig. 8).

The SVF value of Point D being “0” resulted in similar temperatures on opposite surfaces. Furthermore, the temperature differences between morning and afternoon measurements at this point were generally smaller compared to other points. Although Points D1, D2, and D3 had the same SVF values, their surface temperatures differed. This variation can be attributed to additional factors such as street orientation and differences in building height (Fig. 8).

**Fig. 9:**
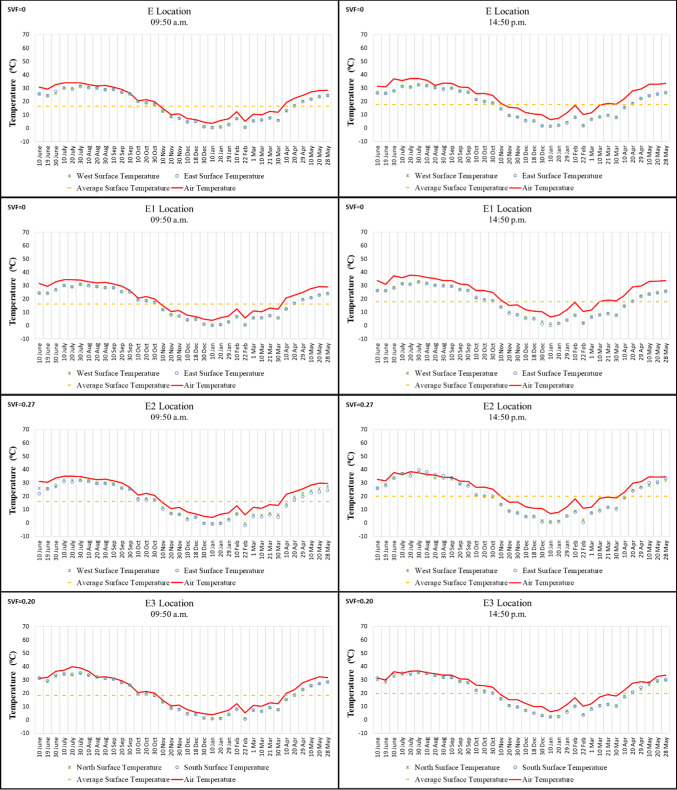
Surface temperature measurement results of location E.

Of the points in Region E, Points E, E1 (the start and end points of the *kabaltı*), and E2 are located on a north–south oriented street, while Point E3 is situated on an east–west oriented street. The lowest surface temperature in the morning was recorded at − 2 °C on 22 February 2021 on the east wall of Point E2, while the highest was 35.3 °C on 30 July 2020 on the south wall of Point E3. In the afternoon, the highest temperature was measured at 40 °C on 30 July 2020 on the east wall of Point E2, and the lowest was 0.4 °C on 10 January 2021, also on the east wall of Point E2 (Fig. 9).

According to the morning measurements, the four points had similar values, whereas in the afternoon the surface temperature of Point E2 increased by 5–10 °C. For the other points, the temperature variation between morning and afternoon was only 1–2 °C. This small difference is attributed to the low SVF values at these points and the limited exposure of building surfaces to direct solar radiation (Fig. 9).

**Fig. 10:**
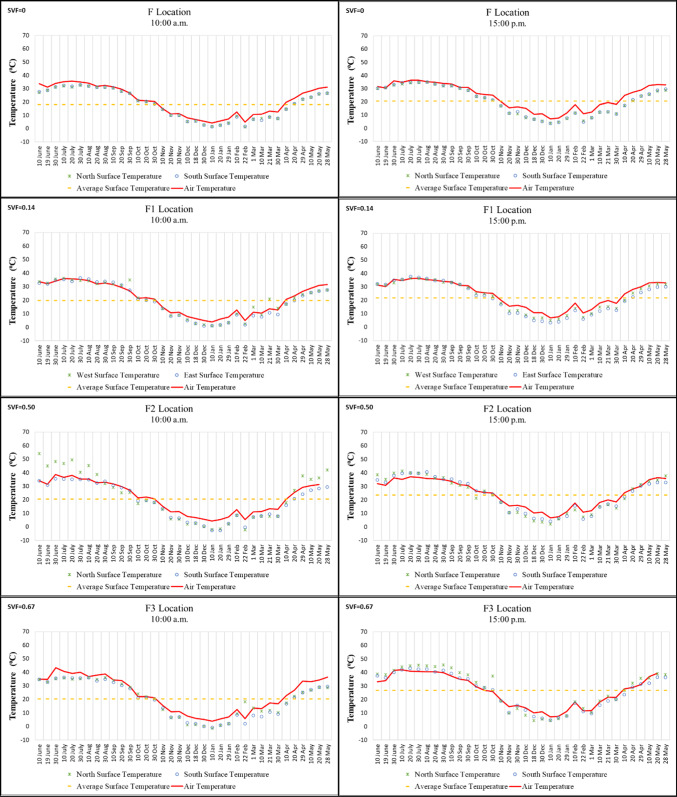
Surface temperature measurement results of location F.

In Region F, Points F, F2, and F3 are located on an east–west oriented street, while Point F1 is situated on a north–south oriented street. In the morning measurements, the highest surface temperature was 54.2 °C on 10 June 2020 on the north wall of Point F2, while the lowest was − 2.6 °C on 10 January 2021 on the south wall of Point F2. In the afternoon measurements, the highest surface temperature was 45.6 °C on 30 August 2020 on the north wall of Point F3, while the lowest was 2.1 °C on 10 January 2021 on the north wall of Point F2 (Fig. 10).

The variations in surface temperatures at Points F and F1 during morning and afternoon hours were very similar. The presence of an overhead cover at Point F1 prevented the building surfaces from being directly exposed to sunlight. Consequently, the temperature fluctuations at F1, which has a low SVF value, were smaller than those observed at the other two points. This finding is consistent with Chen et al.^10^, who reported that shading in areas with low SVF values reduces both surface and air temperatures, particularly at noon. By contrast, the building surfaces of the other points exposed to direct sunlight exhibited temperature changes of approximately 5–15 °C (Fig. 10).

**Fig. 11:**
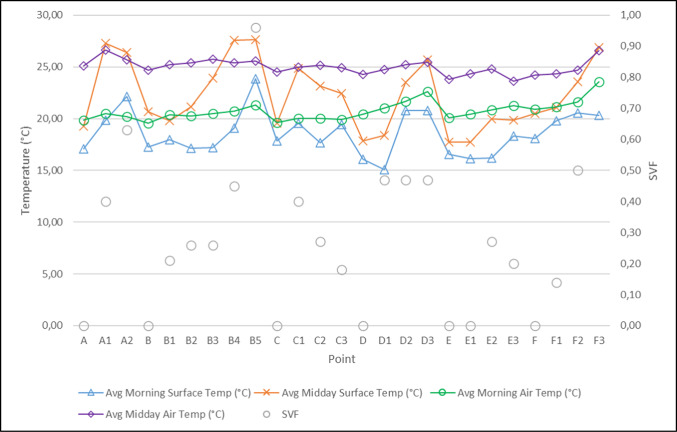
Average morning and midday surface temperatures, air temperatures and SVF values.

**Fig. 12:**
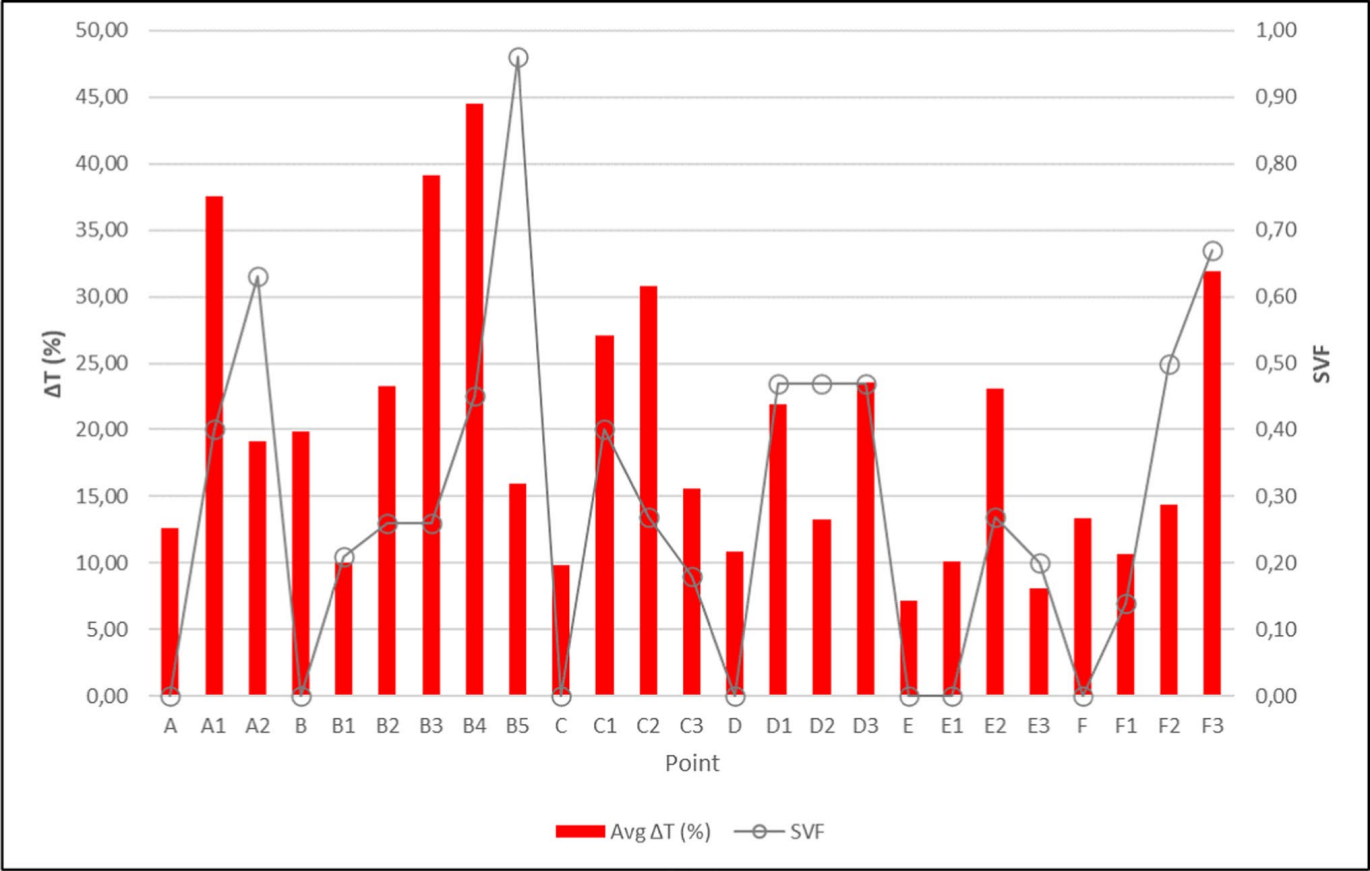
Percentage change in average surface temperatures.

The relationship between the percentage change in average morning and afternoon temperatures at each measurement point and the SVF values was examined. Kabaltıs with an SVF value of 0 exhibited smaller diurnal surface temperature amplitude compared to street arrangements with clear sky visibility; this is attributed to the combined effects of complete sky blockade, reduced direct solar radiation, and limited radiative exchange with the sky. The results show smaller daily surface temperature amplitudes in regions with lower SVF values and in kabaltıs with an SVF value of 0 (Fig. 11, 12) (Table 5).

**Table 5:** Diurnal surface temperature amplitude by street orientation and SVF group.

	SVF Group	Average diurnal surface temperature amplitude (°C)	Range (°C)
West–East	Low SVF (≤ 0.20)	2.56	1.62–3.43
	High SVF (> 0.20)	5.19	3.75–8.50
North–South	Low SVF (≤ 0.20)	1.97	1.75–2.41
	High SVF (> 0.20)	4.21	1.81–6.50

Table 5 compares daily surface temperature amplitudes for two street directions (West–East, North–South), grouped according to low (SVF ≤ 0.20) and high (SVF > 0.20) sky visibility factor values. Values are reported for descriptive comparison purposes based on measured surface temperature data. Although many environmental parameters affect surface temperature, keeping the street orientation constant allowed for the investigation of the relative effect of SVF on the daily surface temperature amplitude under comparable geometric conditions.

## Conclusions

Since the surface temperatures of the points with kabaltıs and SVF values of “0” (A, B, C, D, E, E1, F) are not exposed to direct sunlight during the day, the difference between morning and noon measurements was ranged 2–5 °C. At points with low SVF values (i.e., close to 0), the surface temperatures were measured close to, or in some cases lower than, those of the kabaltı when shading was intense (e.g., D-D1, C-C2). During the hours when these points were exposed to the sun, their surface temperatures increased by approximately 5–10 °C compared to the kabaltı. By contrast, at points with SVF values close to 1, surface temperatures varied by approximately 15–20 °C between shaded and sunlit periods. In addition, extremely high temperatures were measured at these points, such as 58.8 °C at Point B5 and 45 °C at Point F3.

The evaluation of SVF and building surface temperatures indicates that while these two parameters influence one another, they are not entirely interdependent. Urban geometry, including building heights and height differences, orientations, and street widths, also plays a critical role in determining the amount of solar radiation received by buildings and their surface temperatures. This is evident from differences in surface temperatures at points shaped by different street geometries but with similar SVF values (e.g., B4-D2, B3-E2, D1-D3). Large building height differences were shown to increase solar exposure and, consequently, temperature differences. Similarly, wider streets were found to increase SVF values and surface temperatures. Furthermore, the shading effects created by surrounding buildings of varying heights also influenced street-level temperatures.

When comparing building surface temperatures within each region, both between kabaltıs and points with the highest SVF, the kabaltıs consistently exhibited much lower values (e.g., A-A2, B-B5, C-C1, D-D3, F-F3). Across both street orientations, kabaltıs consistently exhibited lower surface temperatures than the most exposed street segments with high SVF values. Measured diurnal surface temperature amplitudes at SVF = 0 locations were typically below 3 °C, whereas amplitudes at high-SVF locations reached approximately 4–8 °C, corresponding to increases of nearly 100% depending on street orientation. These results indicate that reduced sky exposure significantly decreases daytime surface temperature fluctuations. The moderating effect observed at SVF = 0 locations (kabaltıs) can be attributed to persistent shading, which limits direct solar radiation and reduces rapid surface heating during daytime hours. The findings in this study are consistent with those of previous studies on street geometry and SVF. Ali-Toudert and Mayer^34^ and Johansson^35^ showed that narrow streets with lower SVF values resulted in lower wall surface temperatures in hot-dry climates, similar to the shaded kabaltıs in this study. Similar to the findings in this study, Deng and Wong^36^ found that higher SVF values increased solar radiation exposure and confirmed the correlation between SVF and surface temperature. Chen et al.^10^ emphasized that shading and vegetation reduced wall surface temperature during high solar radiation hours, supporting the results obtained for the kabaltıs in this study. Consistent with Aboelata^37^, the results obtained in this study suggest that integrating shading strategies and vegetation into the streetscape can improve outdoor comfort. To create comfortable urban environments, strategies should therefore focus on significantly reducing solar radiation reaching building surfaces, particularly during the daytime hours of hot seasons, while also enhancing surface cooling effects.

While kabaltıs can reduce surface temperatures during morning hours through shading, they also have potential disadvantages. A low SVF in kabaltıs can have an undesirable effect by causing heat to be retained for longer periods at night. Consequently, air temperatures can be relatively higher in kabaltıs at night compared to open streets. Furthermore, the semi-open space nature of kabaltıs can potentially weaken airflow. This negative potential suggests that, despite the advantages of kabaltıs during daylight hours, their microclimatic effects should be investigated with a comprehensive approach.

This study highlights the importance of kabaltıs and their shading and microclimatic functions within the traditional Suriçi settlement of Diyarbakır, a city located in a hot-dry climate. The existing kabaltıs should be preserved to maintain their beneficial microclimatic properties, while damaged structures should be restored through appropriate conservation interventions. Preserving these traditional features will not only mitigate the negative impacts of the local climate but will also add cultural value by maintaining the historical and architectural integrity of the settlement.

The findings of this study point to a fundamental principle influencing the thermal behavior of urban surfaces beyond the local case context. The results show that street orientation and sky openness are among the key factors determining the thermal variability of urban surfaces throughout the day. This finding offers broader conceptual implications for understanding surface temperature variations in different urban contexts.

### Limitations and future studies

This study is limited to one year of field measurements and does not include the effects of wind speed, humidity, or material changes. Therefore, the results reflect the specific conditions of the traditional Suriçi area and may not be directly representative of other urban settlements. Future research could expand on the impact of different climatic zones, longer measurement periods, street geometry, and different basement typologies on thermal comfort and energy performance.

## Data Availability

The data supporting the findings of this study can be accessed from corresponding author upon reasonable request.
